# High levels of *Bifidobacteriaceae* are associated with the pathogenesis of Parkinson’s disease

**DOI:** 10.3389/fnint.2022.1054627

**Published:** 2023-01-04

**Authors:** ShuJia Zuo, HaiJing Wang, Qiang Zhao, Jie Tang, Min Wang, Yu Zhang, Ming Sang, Jing Tian, Puqing Wang

**Affiliations:** ^1^Postgraduate Union Training Base of Jinzhou Medical University, Xiangyang No.1 People’s Hospital, Hubei University of Medicine, Xiangyang, Hubei, China; ^2^Department of Neurology, Xiangyang No.1 People’s Hospital, Hubei University of Medicine, Xiangyang, Hubei, China; ^3^Hubei Clinical Research Center of Parkinson’s Disease, Xiangyang Key Laboratory of Movement Disorders, Xiangyang No.1 People’s Hospital, Hubei University of Medicine, Xiangyang, Hubei, China

**Keywords:** gut microbiota, meta-analysis, Parkinson’s disease, KEGG pathway, 16S rRNA

## Abstract

**Background:**

The diagnosis of Parkinson’s disease (PD) is complex and there are no biomarkers for early identification. Many studies have reported altered gut microbiota in patients with PD compared with healthy individuals. However, results from previous studies vary across countries.

**Aims:**

The aim of this study was to identify gut microbiota biomarkers that could be used as a marker for the diagnosis of PD.

**Methods:**

Firstly, the differential gut microbiota was obtained by meta-analysis, and then the results of meta-analysis were validated through metagenomic cohort. Finally, the ROC curve was drawn based on the metagenomic validation results.

**Results:**

The meta-analysis showed a lower relative abundance of *Prevotellaceae* (*p* < 0.00001) and *Lachnospiraceae* (*p* = 0.002), and a higher of *Ruminococcaceae* (*p* < 0.00001), *Christensenellaceae* (*p* = 0.03), *Bifidobacteriaceae* (*p* < 0.00001), and *Verrucomicrobiaceae* (*p* = 0.23) in patients with PD. Only *Bifidobacteriaceae* was also at high levels in the validation cohort of the metagenome. Meanwhile, three species from the *Bifidobacteriaceae*, including *Scardovia_inopinata* (*p* = 0.022), *Bifidobacterium_dentium* (*p* = 0.005), and *Scardovia_wiggsiae* (*p* = 0.024) were also high. The ROC curve showed that the three species (71.2%) from *Bifidobacteriaceae* had good predictive efficiency for PD.

**Conclusion:**

Elevated *Bifidobacteriaceae* may be associated with PD. Elevated three species from the *Bifidobacteriaceae*, including *Scardovia_inopinata*, *Bifidobacterium_dentium* and *Scardovia_wiggsiae* may provide new potential biomarkers for the diagnosis of PD.

## Introduction

Parkinson’s disease (PD) is the second most common neurodegenerative disease, characterised by clinical manifestations of resting tremor, bradykinesia, and/or muscle stiffness (Liddle, 2018; Fayyad et al., 2019). Currently, the diagnosis of PD depends largely on clinical manifestation scales, imaging examination and the experience of clinicians (Su et al., 2020). However, the scales are often subjective and easily affected by symptoms. Functional neuroimaging is very expensive and can only be done at a few centers (Bhidayasiri and Martinez-Martin, 2017). It is urgent to find a reliable and economical biomarker for the early diagnosis of PD.

Gastrointestinal dysfunction, particularly constipation, is the most common non-motor symptom of PD and often precedes the motor disability onset by decades (Lin et al., 2019). Similarly, accumulation of alpha-synuclein (α-syn) in the enteric nervous system (ENS) may begin 20 years before the onset of degenerative changes in the central nervous system (CNS) and motor symptoms associated with PD (Hasegawa et al., 2015). Previous studies on pathological changes in the intestines of patients and animal models of PD suggest the possibility that gut alteration is associated with PD pathogenesis (Choi et al., 2018). Thus, we speculate that α-syn appears in the ENS first, and then can be transferred to the CNS.

Recent studies have revealed that gut microbiota and their metabolites affect brain function through neural, endocrine, and immune pathways (Cryan and Dinan, 2012). Meanwhile, the body also regulates the composition of the gut microbiota through this pathway to maintain the balance of intestinal microecology–that is, the microbiota–gut-brain axis (Elfil et al., 2020). The gut microbiota widely participates in the synthesis/release of various hormones and gut-brain axis–related neurotransmitters, which then regulates brain function and host behaviour. In summary, changes in the gut microbiota composition and function play an important role in the occurrence and progression of PD. Researchers have found differences in the gut microbiota composition between patients with PD and healthy controls (HC), but the results are not consistent. For example, Scheperjans et al. (2015) pointed to a relatively lower abundance of *Prevotellaceae* in advanced PD. Bedarf et al. (2017) found *Prevotellaceae* was markedly lower in patients with early-stage PD. However, Keshavarzian et al. (2015) found that there was no statistically significant difference between PD and HC in *Prevotellaceae*.

In addition to our previously published studies on 16S rRNA (Zhang et al., 2020), we included more studies and used meta-analysis to preliminarily determine the altered gut microbiota between PD and HC. Furthermore, metagenomics was used to validate the differential gut microbiota in the meta-analysis results. The possible pathogenic mechanisms of these differential gut microbiota in PD are also discussed.

## Materials and methods

### Meta-analysis

#### Literature retrieval, inclusion, and exclusion

To conduct a meta-analysis of studies related to the gut microbiota in PD, we conducted a comprehensive and systematic literature search using the following English and Chinese databases (for paper published up to October 2021): PubMed, Web of Science, the Chinese National Knowledge Infrastructure (CNKI) databases and the Wanfang database. The search strategy to identify all potential studies involved using combinations of the following terms: (Parkinson’s disease OR Parkinson disease OR Parkinsonism) AND (microbes OR microbiome OR microbiota OR bacteria) in the Title/Abstract. We also searched the articles referenced in the included articles to identify any studies we may have missed with the above-mentioned search strategy. For the articles retrieved through the above-described search strategy, we included studies that met the following criteria: (1) the study was a randomised controlled trial (RCT) comparing differences in the intestinal flora of patients with PD and HCs; (2) the study used faecal samples; (3) the abundance of the microbiome was expressed as the average proportion of each microbiome; (4) differences between patients with PD and HCs were presented with the mean difference (MD) and the 95% confidence interval (CI); and (5) studies with scores > 5 stars as assessed by the nine-star Newcastle–Ottawa Quality Assessment Scale (NOS). We excluded studies with the following criteria: (1) studies without assessment of the measurement indexes; (2) only patient studies, family-based studies, intervention studies and review articles; and (3) studies with scores below five as assessed by the NOS; (4) studies were not considered or explicitly excluded participants who received antibiotics and probiotics 1–3 months before the trial.

#### Data extraction

We extracted the following data from the included studies: (1) general basic information (author, publication date, and location); (2) characteristics of patients with PD and HCs (gender and age); (3) diagnostic criteria, sample sizes, and microbiological experimental methods (detection); and (4) microbiome abundance effect size (MD and 95% CI); (5) clinical features of PD patients (UPDRS total score, score of UPDRS Part III and H&Y stage).

#### Quality assessment

Disagreements were solved through discussion or involvement of a third investigator, if necessary. We included studies with five or more stars after a general evaluation with the NOS in the meta-analysis. Detailed criteria are provided in Supplementary Table 1 (Stang, 2010).

#### Statistical analysis

At least five studies of the same gut microbiota have reported differences between PD and HC. Meta-analysis was conducted using Review Manager Version 5.3 (Cochrane collaboration) to generate forest plots and funnel plots. We calculated mean difference (MD) and 95% confidence interval (CI) of microbiota abundance as summary statistics and generated forest plots. At the same time, the degree of heterogeneity was evaluated by the inconsistency index (*I*^2^) test. The fixed-effects model was used when *I*^2^ < 50%; otherwise, the random-effects model was applied. Heterogeneity is represented by funnel plots. Differences were considered statistically significant when the FDR corrected *p*-value was <0.05.

### Validation of differential gut microbiota

Fecal samples from 39 PD patients and corresponding 39 healthy spouses were collected for shotgun metagenomic sequencing. PD patients were diagnosed according to the 2015 PD diagnostic criteria of the MDS (Li J. et al., 2017). The inclusion criteria of healthy spouses were healthy individuals without PD symptoms. All participants who received or injected antibiotics or probiotics within the last 3 months were further excluded. SPSS (version 25) was used for statistical analysis. First, the relative abundance of gut microbiota was extracted from the metagenomic data. Comparison of two independent groups was performed by Student *t*-test for normally distributed data, and Mann-Whitney U-criteria (M-U test) for not normally distributed data. The results of the meta-analysis were then verified. Finally, SPSS software was used to draw receiver operating characteristic (ROC) curve.

## Results

**FIGURE 1:**
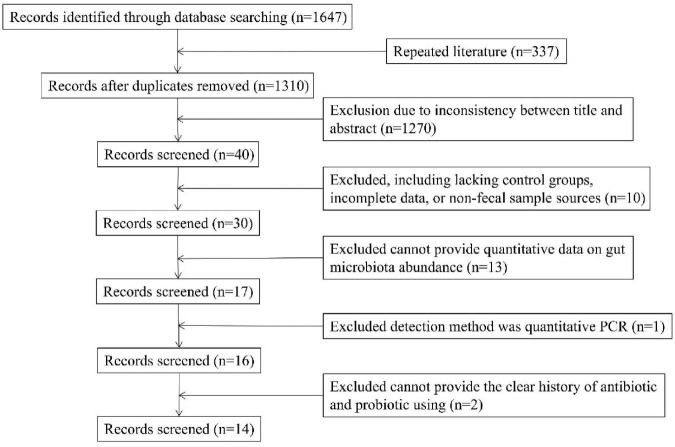
Flow diagram of the assessment of studies used in the meta-analysis.

The literature screening process is summarised as a flow chart (Figure 1). We retrieved 1,647 potential records, of which we excluded 337 duplicates. In addition, we excluded 1,270 studies whose content was inconsistent with our study, 10 studies that lacked control groups, had incomplete data, or used non-fecal samples, 15 studies that could not provide quantitative data on gut microbiota abundance, 2 studies that did not specify exclusion criteria for the use of antibiotics and probiotics (Specific inclusion and exclusion criteria for inclusion studies are in Supplementary Table 2), and 1 study conducted quantitative PCR detection technique. Hence, we included 14 studies in the meta-analysis. The main features of these studies are summarised in Table 1. Based on the NOS scale, all 14 case-control studies are high quality (Supplementary Table 1).

**TABLE 1:** Characteristics of the 14 studies included in the systematic review.

References	Location	Patients with PD/HCs			Experimental methods	
			
		Number	Mean age ± SD (years)	Female (%)	Sample	Technique
Scheperjans et al., 2015	Finland	72/72	65.3 ± 5.5/ 64.5 ± 6.9	48.6/50.0	Faeces	NGS
Hill-Burns et al., 2017	United States	197/130	68.4 ± 9.2/ 70.3 ± 8.6	33.0/60.8	Faeces	NGS
Barichella et al., 2019	Italy	193/113	67.6 ± 9.7/ 65.9 ± 9.9	40.4/58.4	Faeces	NGS
Aho et al., 2019	Finland	64/64	65.2 ± 5.5/ 64.5 ± 6.9	48.4/50.0	Faeces	NGS
Ren et al., 2020	China	14/13	60.0 ± 9.2/ 63.0 ± 8.8	76.9/28.6	Faeces	NGS
Li C. et al., 2019	China	51/48	62.4 ± 8.2/ 62.2 ± 9.2	37.3/60.4	Faeces	NGS
Li W. et al., 2017	China	24/14	73.8 ± 6.3/ 74.6 ± 5.6	33.3/57.1	Faeces	NGS
Li F. et al., 2019	China	10/10	76.5 ± 7.1/ 79.5 ± 7.6	30.0/50.0	Faeces	NGS
Tan et al., 2021	Malaysia	104/96	65.4 ± 8.4/ 62.4 ± 9.0	37.5/62.5	Faeces	NGS
Zhang et al., 2020	China	63/137	64.0 ± 7.4/ 63.9 ± 7.9	36.5/41.9	Faeces	NGS
Peng et al., 2021	China	70/90	71.9 ± 6.2/ 72.8 ± 5.9	55.7/48.9	Faeces	NGS
Hopfner et al., 2017	Germany	29/29	69.2 ± 6.5/ 69.4 ± 6.7	20.7/55.2	Faeces	NGS
Bedarf et al., 2017	Germany	31/28	64.8 ± 9.5/ 65.6 ± 10.4	NA	Faeces	NGS
Lin et al., 2018	China	75/45	60.5 ± 10.7/ 63.2 ± 6.0	34.7/48.9	Faeces	NGS

HC, healthy controls; NA, not provided; NGS, next-generation sequencing; PD, Parkinson’s disease; SD, standard deviation.

A total of 1,886 subjects were included in the meta-analysis, 997 were PD patients (age range: 60.0–76.5 years; percent of female subjects ranged: 20.7–76.9; H&Y: 1–3; UPDRS-III score: 12.6–33.7; UPDRS total score: 30.5–49.0) and 889 were healthy controls (age: 62.4–79.5 years; percent of female subjects ranged: 28.6–62.5). All 14 studies had detected gut microbiota by next-generation sequencing (NGS) (see Table 1 for the demographic data).

### Meta-analysis of standardised MD

**FIGURE 2:**
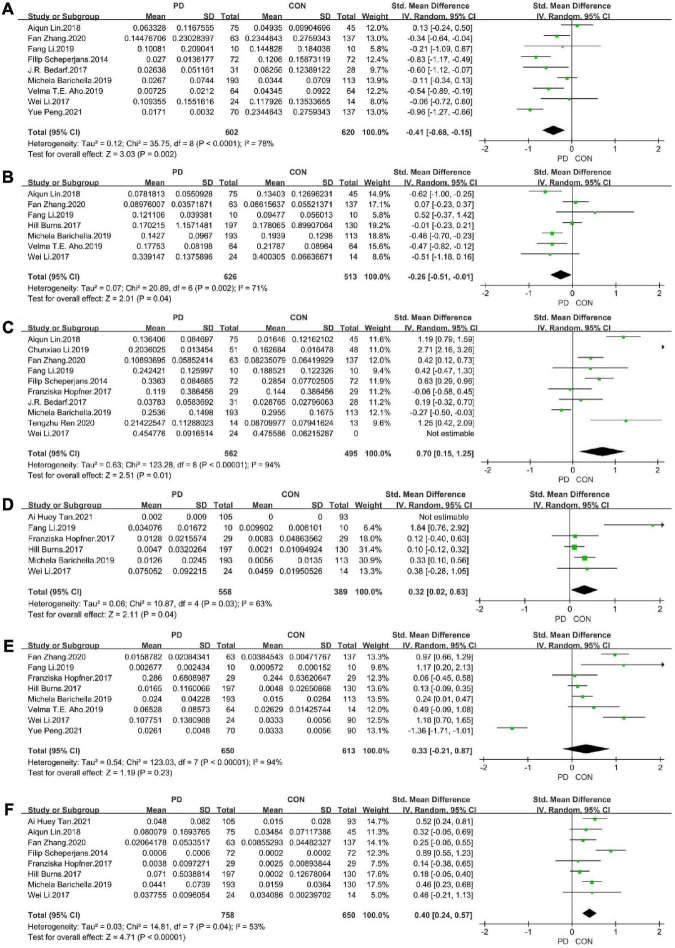
Forest plots of alterations in gut microbiota in patients with Parkinson’s disease (PD) compared with healthy controls (HC). **(A)** f_*Prevotellaceae*, **(B)** f_*Lachnospiraceae*, **(C)** f_*Ruminoccoccaceae*, **(D)** f_*Christensenellaceae*, **(E)** f_*Verrucomicrobiaceae*, and **(F)** f_*Bifidobacteriaceae*.

**FIGURE 3:**
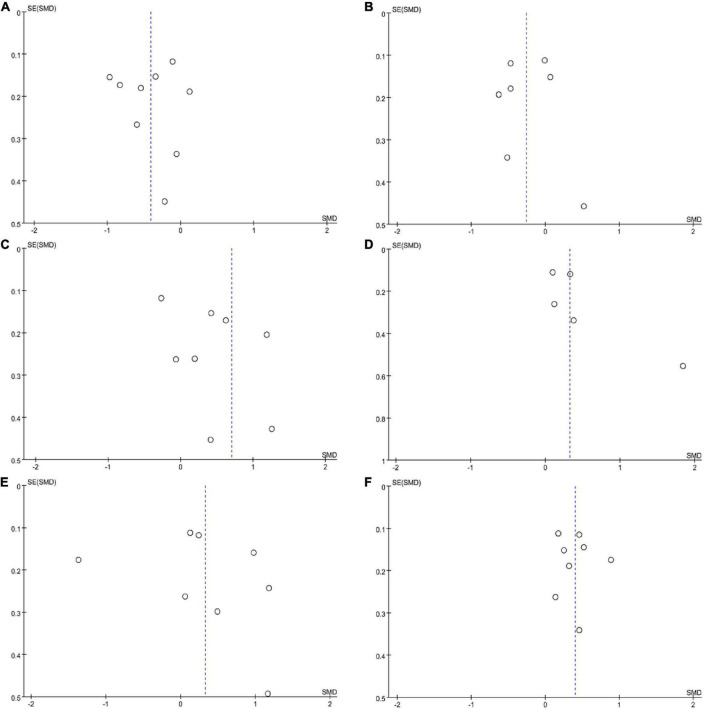
Funnel plots of alterations in gut microbiota in patients with Parkinson’s disease (PD) compared with healthy controls (HCs). **(A)** *Prevotellaceae*, **(B)** *Lachnospiraceae*, **(C)** *Ruminoccoccaceae*, **(D)** *Christensenellaceae*, **(E)** *Verrucomicrobiaceae*, and **(F)** *Bifidobacteriaceae*.

We extracted continuous data from the 14 included studies for the meta-analysis. We analysed alterations in the abundance of f_*Prevotellaceae*, f_*Bifidobacteriaceae*, f_*Lachnospiraceae*, f_*Ruminococcaceae*, f_*Verrucomicrobiaceae*, and f_*Christensenellaceae* in patients with PD. The meta-analysis revealed lower abundance of f_*Prevotellaceae* (MD = –0.41, 95% CI = –0.68 to –0.15; *I*^2^ = 78%; *p* < 0.00001; 9 studies, Figures 2A, 3A) and f_*Lachnospiraceae* (MD = –0.26, 95% CI = –0.51 to –0.01; *I*^2^ = 71%; *p* < 0.0001; 7 studies, Figures 2B, 3B) in patients with PD compared with HCs. On the other hand, there was higher abundance of f_*Ruminococcaceae* (MD = 0.70, 95% CI = 0.15 to 1.25; *I*^2^ = 94%; *p* < 0.00001; 10 studies, Figures 2C, 3C), f_*Christensenellaceae* (MD = 0.32, 95% CI = 0.02 to 0.63; *I*^2^ = 63%; *p* = 0.03; 6 studies, Figures 2D, 3D), f_*Bifidobacteriaceae* (MD = 0.33, 95% CI = –0.21 to 0.87; *I*^2^ = 94%; *p* < 0.00001; 8 studies, Figures 2F, 3F) and f_*Verrucomicrobiaceae* (MD = 0.40, 95% CI = 0.24 to 0.57; *I*^2^ = 53%; *p* = 0.23; 8 studies, Figures 2E, 3E) in patients with PD compared with HCs.

### Validation of the six differential gut microbiota in the above meta-analysis results

We extracted the differential family level gut microbiota and found that among the six different microbiota obtained by meta-analysis, only *Bifidobacteriaceae* was also at high levels in the validation cohort of the metagenome. And three species from f_*Bifidobacteriaceae* included s_*Scardovia_inopinata*, s_*Bifidobacterium_dentium*, and s_*Scardovia_wiggsiae* were also high (Table 2).

**TABLE 2:** Altered families, genera, and species in the metagenomic validation cohort.

Taxa	Name	PD	HP	*Z-*value	*P*-value
Family	f__Bifidobacteriaceae	0.44158 (0.03316, 1.57324)	0.14397 (0.02678, 0.36334)	–1.994	0.046
Species	s__Scardovia_inopinata	0.000 (0.000, 0.000)	0.000 (0.000, 0.000)	–2.295	0.022
	s__Bifidobacterium_dentium	0.0017 (0.000, 0.07531)	0.000 (0.000, 0.00275)	–2.804	0.005
	s__Scardovia_wiggsiae	0.00017 (0.000, 0.00564)	0.000 (0.000, 0.00126)	–2.256	0.024

The statistical method used was the M-U test. Measurement data did not meet the normal distribution, and were represented as M (P_25_, P_75_).

**FIGURE 4:**
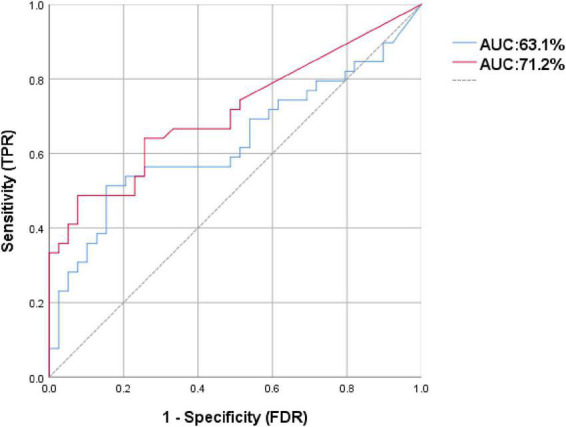
The receiver operating characteristic (ROC) curve for predicting the occurrence of Parkinson’s disease (PD) in 78 samples: The dotted line represents the performance of the probabilistic model, the blue line represents the classification performance of f_*Bifidobacteriaceae*, the red line represents the classification performance of the s_*Scardovia_inopinata*, s_*Bifidobacterium_dentium*, and s_*Scardovia_wiggsiae*.

We used the abundance of f_*Bifidobacteriaceae* and the abundance of s_*Scardovia_inopinata*, s_*Bifidobacterium_dentium*, and s_*scardovia_wiggsiae* generated ROC curves separately (Figure 4). The AUC were 63.1 and 71.2%, respectively. The ROC curve showed that the three species (71.2%) from f_*Bifidobacteriaceae* had good predictive efficiency for PD.

## Discussion

**FIGURE 5:**
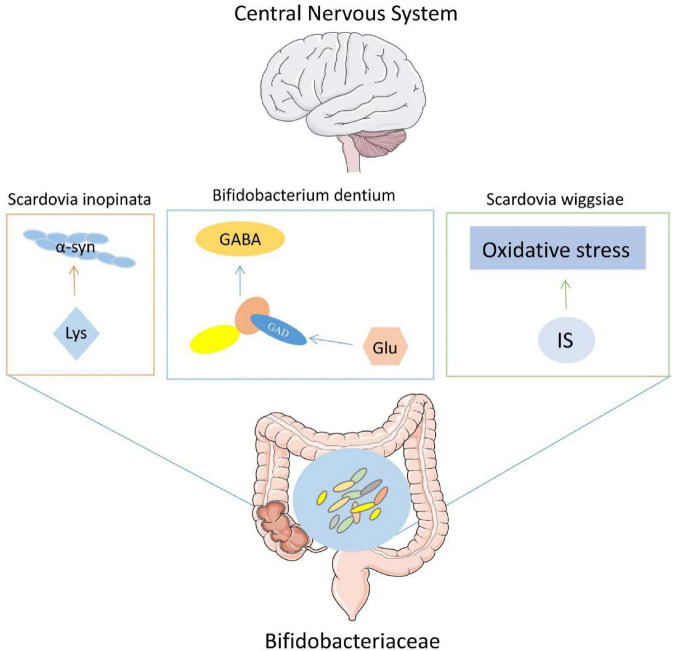
An overview of the pathogenesis of three species from *Bifidobacteriaceae* in Parkinson’s disease (PD).

We included 14 case-control studies in this meta-analysis and found the abundance f_*Prevotellaceae*, f_*Lachnospiraceae* were reduced, the abundance f_*Bifidobacteriaceae*, f_*Ruminococcaceae*, f_*Verrucomicrobiaceae*, and f_*Christensenellaceae* were elevated in PD. Furthermore, additional metagenomic verification showed that only f_*Bifidobacteriaceae* is higher in PD. At the same time, three species from f_*Bifidobacteriaceae* also showed a high abundance, including s_*Scardovia_inopinata*, s_*Bifidobacterium_dentium*, and s_*Scardovia_wiggsiae* (Figure 5).

F_*Bifidobacteriaceae* is considered to be one of the important probiotics that participates in several protective physiological functions. Whereas, 8 studies in the meta-analysis consistently reported that patients with PD have increased abundance of f_*Bifidobacteriaceae*. It suggests that the elevated of f_*Bifidobacteriaceae* in PD may not be an accidental phenomenon. We speculate that some species from f_*Bifidobacteriaceae* may have played a potential promoting role in the pathogenesis of PD.

Protein functional analysis was performed using the UniProt Knowledgebase (UniProtKB) at UniProt.^1^ The search found s_*Scardovia_inopinata* may have the function of aspartate-semialdehyde dehydrogenase (Homologous gene: asd, 373 amino acids), which enzyme is one of the nine enzymes present in microbiota responsible for lysine biosynthesis. All is known that the accumulation of α-syn in the brain forms Lewy body, which leads to the death of dopaminergic neurons (DN) and is thought to be one of the important causes of PD. In fact, the protein sequence of α-syn has a remarkable amount of lysine (Shi et al., 2019). Likewise, not only in the brain, but also in the presence of α-syn in ENS. Therefore, based on the possible function of s_*Scardovia_inopinata*, we speculate that the aggregation of α-syn in ENS may come from the lysine produced by s_*Scardovia_inopinata*, which is further converted aldehydes.

In PD, in addition to the progressive degeneration of the substantia nigra pars compacta and other pigmented nuclei of the brain (Khan et al., 2019). Studies (Sanjari et al., 2017) have shown that gamma – aminobutyric acid ergic (GABAergic) and other neurotransmitter systems also have dysfunction in PD. A study on s_*Bifidobacterium_dentium* found that s_*Bifidobacterium_dentium* can converted glutamate to GABA by the enzyme glutamate decarboxylase [GAD; Enzyme Commission number (EC) 4.1.1.15] (Luck et al., 2021). s_*Bifidobacterium_dentium* not only secretes GABA *in vitro* microbial medium, but also increases the level of GABA in the stool of mice treated with s_*Bifidobacterium_dentium* compared with the bacteria-free control group. Clinical studies have found that increased levels of GABA correlate with the degree of gait disorder in PD (Siucinska, 2019). Therefore, we speculated that s_ *Bifidobacterium_dentium* could promote PD by increasing the level of GABA. The study also found that in addition to s_*Bifidobacterium_dentium*, thirty out of 83 *Bifidobacteria* species contained succinate-semialdehyde dehydrogenase (EC 1.2.1.16) for converting succinate to GABA. This may explain why f_*Bifidobacteriaceae*, known as probiotics, promote PD.

Finally, in a study investigating the relationship between serum inflammatory factors and gut microbiota in PD with end-stage renal disease, s_*Scardovia_wiggsiae* was found to have a significant positive correlation with the inflammatory factor indole sulfate (IS) (Asgharian et al., 2022). A study on the concentration of toxins in the blood and cerebrospinal fluid of PD patients found that IS also increased and affected the development of PD through inflammation and oxidative stress (Sankowski et al., 2020). Recent studies have suggested that oxidative stress plays an important role in the process of neurodegeneration in PD. We speculated that the increase of s_*Scardovia_wiggsiae* could lead to the increase of IS level in blood and cerebrospinal fluid and aggravate the degree of oxidative stress, which may lead to the occurrence of PD. Furthermore, the ROC curve showed that the three species (71.2%) from *Bifidobacteriaceae* had good predictive efficiency for PD.

In addition to diseases, studies have shown that physiological aging is related to intestinal ecological disorders. A study to investigate the impact of aging on the gut microbiota and to probe the bacterial taxonomic composition of fecal samples from younger (25–49 years) and older (61–100 years) persons showed that the f_*Bifidobacteriaceae*, known as probiotics, showed a decrease in abundance with age (Mueller et al., 2006). These results suggest that the protective mechanisms of human body gradually decline with aging, which leads to the decrease of beneficial microbiota and the increase of harmful microbiota. Therefore, its elevated in PD further indicates the potential pathogenic effect of f_*Bifidobacteriaceae*, but the specific mechanism needs further study. Due to several factors, including the delivery mode, feeding during infancy, eating habits, culture, geographical area, age and gender, among others, the structure of intestinal microflora varies among individuals (Zhuang et al., 2017). However, even taking these factors into account, we found common changes in the abundance of f_*Bifidobacteriaceae* in patients with PD across different studies conducted in different geographical regions. Thus, it is highly likely that these alterations in the gut microbiota result from PD itself.

## Limitations

Our meta-analysis had some limitations. First, there was statistical bias among the included studies, as the majority of the subjects included were patients with PD who came to the hospital for medical treatment and physical examination. Second, we discussed only the structure and composition of the gut microbiota, but transcriptomic and proteomic studies would provide greater insight into the function of the gut microbiota. Third, all the included studies used 16S rRNA amplicon sequencing techniques to analyse the diversity of the gut microbiota. However, 16S rRNA primers for different regions may result in inconsistent results because their corresponding flanking conservative regions not only have a significant binding affinity, but also differ in the resolution of each variable region in the taxon, resulting in deviations due to the detection technique. Finally, we extracted only MD and 95% CI from the studies and did not analyse raw data to eliminate the method biases. These factors need to be improved in future studies.

## Conclusion

Our meta-analysis identified six differential gut microbiota in PD patients. Through validation, only *Bifidobacteriaceae* was also found to have high levels in the metagenomics validation cohort. Meanwhile, three species from the *Bifidobacteriaceae*, including *Scardovia_inopinata*, *Bifidobacterium_dentium*, and *Scardovia_wiggsiae* were also high. Our findings suggest that high levels of *Bifidobacteriaceae* may be involved in the pathogenesis of PD. The high levels of three species from Bifidobacteriaceae in patients may affect PD by participating in the aggregation of α-syn, the increase of GABA and IS levels. The three species (71.2%) from *Bifidobacteriaceae* had good predictive efficiency for PD. In the future, interventions of the above gut microbiota may help to prevent PD and improve disease progression.

## Data availability statement

Publicly available datasets were analyzed in this study. This data can be found here: 16S rRNA sequence data from the Genome Sequence Archive (CRA001938); metagenomic study (PRJNA588035).

## Ethics statement

The studies involving human participants were reviewed and approved by Ethics Committee of Xiangyang No.1 People’s Hospital. The patients/participants provided their written informed consent to participate in this study.

## Author contributions

PW and JTi: conceptualisation. JTi: methodology. QZ: software, data curation, and funding acquisition. JTa and HW: validation. YZ: formal analysis. SZ and HW: investigation. PW: resources, writing – original draft preparation, writing – review and editing, supervision, and project administration. MS: visualisation. All authors contributed to the article and approved the submitted version.

## Footnotes

1
http://www.uniprot.org

## Supplementary material

Supplementary Table 1 Scores of the 14 studies included in this meta-analysis based on the Newcastle–Ottawa Quality Assessment Scale (NOS).

Supplementary Table 2 Inclusion and exclusion criteria for the 14 studies included in this meta-analysis.
