# Corrigendum: High levels of *Bifidobacteriaceae* are associated with the pathogenesis of Parkinson's disease

**DOI:** 10.3389/fnint.2023.1229949

**Published:** 2023-07-17

**Authors:** ShuJia Zuo, HaiJing Wang, Qiang Zhao, Jie Tang, Min Wang, Yu Zhang, Ming Sang, Jing Tian, Puqing Wang

**Affiliations:** ^1^Postgraduate Union Training Base of Jinzhou Medical University, Xiangyang No.1 People's Hospital, Hubei University of Medicine, Xiangyang, Hubei, China; ^2^Department of Neurology, Xiangyang No.1 People's Hospital, Hubei University of Medicine, Xiangyang, Hubei, China; ^3^Hubei Clinical Research Center of Parkinson's Disease, Xiangyang Key Laboratory of Movement Disorders, Xiangyang No.1 People's Hospital, Hubei University of Medicine, Xiangyang, Hubei, China

**Keywords:** gut microbiota, meta-analysis, Parkinson's disease, KEGG pathway, 16S rRNA

In the published article, there was an error in the legend for “Figure 2. Forest plots of alterations in gut microbiota in patients with Parkinson's disease (PD) compared with healthy controls (HC) and Figure 3. Funnel plots of alterations in gut microbiota in patients with Parkinson's disease (PD) compared with healthy controls (HCs)” as published. E is written as *Bifidobacteriaceae* and F is written as *Verrucomicrobiaceae* in the figure legend. The corrected legend appears below.

“E should be f_*Verrucomicrobiaceae* and F should be f_*Bifidobacteriaceae* in the Figure 2 legend; E should be *Verrucomicrobiaceae* and F should be *Bifidobacteriaceae* in the Figure 3 legend.”

Figure 2. Forest plots of alterations in gut microbiota in patients with Parkinson's disease (PD) compared with healthy controls (HC). **(A)** f_*Prevotellaceae*, **(B)** f_*Lachnospiraceae*, **(C)** f_*Ruminoccoccaceae*, **(D)** f_*Christensenellaceae*, **(E)** f_*Verrucomicrobiaceae*, and **(F)** f_*Bifidobacteriaceae*.

Figure 3. Funnel plots of alterations in gut microbiota in patients with Parkinson's disease (PD) compared with healthy controls (HCs). **(A)** *Prevotellaceae*, **(B)** *Lachnospiraceae*, **(C)** *Ruminoccoccaceae*, **(D)** *Christensenellaceae*, **(E)** *Verrucomicrobiaceae*, and **(F)** *Bifidobacteriaceae*.

In the published article, there was an error. The *P*-value of *Verrucomicrobiaceae* was incorrectly written.

A correction has been made to **Abstract**, “*Results*,” [1]. This sentence previously stated:

“*Verrucomicrobiaceae* (*p* = 0.02)”

The corrected sentence appears below:

“*Verrucomicrobiaceae* (*p* = 0.23)”

In the published article, there was an error. The figure legend *f_Bifidobacteriaceae* and *f_Verrucomicrobiaceae* were written backwards; the *P*-value of *f_Verrucomicrobiaceae* was written wrong.

A correction has been made to **Results**, “*Meta-analysis of standardised MD*,” [1]. This sentence previously stated:

“*f_Bifidobacteriaceae* (MD = 0.33, 95% CI = −0.21 to 0.87; *I*^2^ = 94%; *p* < 0.00001; 8 studies, Figures 2E, 3E) and *f_Verrucomicrobiaceae* (MD = 0.40, 95% CI = 0.24 to 0.57; *I*^2^ = 53%; *p* = 0.02; 8 studies, Figures 2F, 3F) in patients with PD compared with HCs.”

The corrected sentence appears below:

“*f_Bifidobacteriaceae* (MD = 0.33, 95% CI = −0.21 to 0.87; *I*^2^ = 94%; *p* < 0.00001; 8 studies, Figures 2F, 3F) and *f_Verrucomicrobiaceae* (MD = 0.40, 95% CI = 0.24 to 0.57; *I*^2^ = 53%; p = 0.23; 8 studies, Figures 2E, 3E) in patients with PD compared with HCs.”

The authors apologize for this error and state that this does not change the scientific conclusions of the article in any way. The original article has been updated.

