# Supplementary material for: High levels of Bifidobacteriaceae are associated with the pathogenesis of Parkinson’s disease
Source: Front Integr Neurosci. 2023 Jan 4;16:1054627. doi: 10.3389/fnint.2022.1054627 (PMC9846222; doi:10.3389/fnint.2022.1054627)
Supplement: Supplementary Table 1 — Scores of the 14 studies included in this meta-analysis based on the Newcastle–Ottawa Quality Assessment Scale (NOS). [file Table_1.docx]

**Supplementary Table 1: Scores of the 14 studies included in this meta-analysis based on NOS**

| References | Selection | | | | Comparability | Exposure | | | Total Score |
| --- | --- | --- | --- | --- | --- | --- | --- | --- | --- |
|  | Adequate Definition of Cases | Representativeness of Cases | Selection of Controls | Definition of Controls | Control for Important Factor | Ascertainment of Exposure | Same Method to Ascertain for Cases and Controls | Non-response Rate |  |
| Filip Scheperjans.2014 | 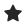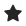 | 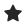 | - | 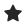 | 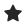 | 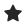 | 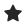 | 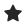 | 7 |
| Hill Burns. 2017 | 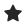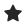 | 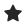 | 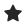 | 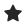 | - | 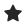 | 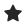 | - | 6 |
| Michela Barichella.2019 | 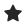 | 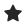 | 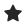 | 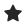 | 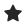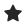 | 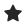 | 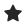 | - | 8 |
| Velma T.E.Aho.2019 | 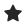 | 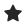 | 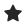 | 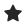 | 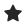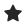 | 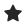 | 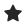 | 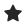 | 9 |
| Tengzhu Ren.2020 | 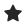 | 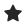 | - | 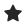 | 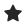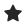 | 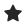 | 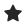 | - | 7 |
| Chunxiao Li.2019 | 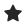 | 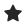 | 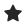 | 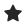 | 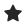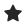 | 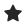 | 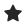 | - | 8 |
| Wei Li.2017 | 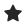 | 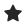 | 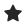 | 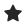 | 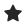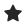 | 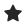 | 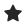 | - | 8 |
| Fang Li.2019 | - | 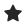 | - | 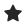 | 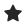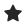 | 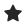 | 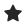 | - | 6 |
| Ai Huey Tan.2021 | 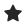 | 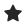 | 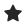 | 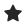 | 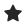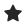 | 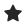 | 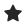 | - | 8 |
| Fan Zhang.2020 | 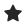 | 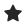 | 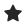 | 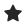 | 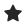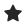 | 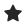 | 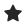 | - | 8 |
| Yue Peng.2021 | 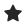 | 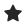 | - | 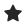 | 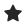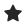 | 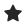 | 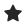 | - | 7 |
| Franziska Hopfner.2017 | 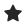 | 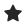 | - | 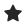 | 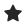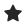 | 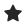 | 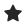 | - | 6 |
| J.R.Bedarf.2017 | 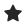 | 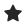 | - | 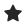 | 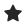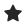 | 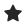 | 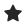 | - | 7 |
| Aiqun Lin.2018 | 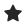 | 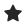 | - |  |  |  |  | - | 6 |
