# Supplementary material for: High levels of Bifidobacteriaceae are associated with the pathogenesis of Parkinson’s disease
Source: Front Integr Neurosci. 2023 Jan 4;16:1054627. doi: 10.3389/fnint.2022.1054627 (PMC9846222; doi:10.3389/fnint.2022.1054627)
Supplement: Supplementary Table 2 — Inclusion and exclusion criteria for the 14 studies included in this meta-analysis. [file Table_2.docx]

**Supplementary Table 2：Inclusion and exclusion criteria for 14 studies**

|  | Inclusion criteria | Exclusion criteria |
| --- | --- | --- |
| 1. Filip Scheperjans.2014 | This case-control study compared patients with a diagnosis of PD （Age of onset > 50 years）according to the Queen Square Brain Bank criteria with sex- and age-matched (65 years) control subjects without any signs of parkinsonism or potential premotor symptoms. | 1. Active smoking within last 6 months; 2.Diagnosis of dementia or MMSE < 25 points; 3.Diagnosis of major depression or GDS-15 > 9 points; 4.Diagnosis of psychosis.   **Environmental or genetic factors：**   1. First degree relative or more than one relative with PD; 2.HIV infection.   **Factors related to or affecting gastrointestinal function：**  1.Active or persistent primary disease of gastrointestinal tract: e.g. celiac disease, pernicious anemia, autoimmune gastritis, symptomatic diverticulosis, inflammatory bowel disease, irritable bowel syndrome, strictures, adhesions, varicosis or diverticulum of the esophagus, Meckel’s diverticulum (Exception: other forms of chronic gastritis); 2.Endocrinological disease (Exception: diabetes mellitus without polyneuropathy; treated hypothyreosis with normal thyrotropin level) ; 3.Alcohol abuse; 4.B-hypovitaminosis; 5.History of hepatobiliar or pancreatic disease (Exception: asymptomatic cholecystolithiasis) ; 6.Previous abdominal or anorectal surgery (Exceptions: can be enrolled after uncomplicated haemorrhoid procedure and 1 year after uncomplicated appendectomy, inguinal hernia repair, cholecystectomia, or gynecologial surgery if symptoms resolved and no signs of adhesions) ; 7.Severe gynaecological prolapse (grade III) (Exception: can be enrolled after repair procedure if symptoms resolved and no signs of adhesions); 8.History of peritonitis, severe endometriosis, polyneuropathy, polio, spina bifida, severe symptomatic spinal stenosis (if not symptom free for at least 1 year), paraparesis of any cause, symptomatic peripheral arteriosclerosis, any signs or history of intestinal ischemia (e.g. claudication), aortic aneurysm or dissection, connective tissue disease, autoimmune disease, sarcoidosis outside of the lungs or skin, cancer that is not considered cured, abdominal, intestinal, or urogenital fistula (Exceptions: treated autoimmunehypothyreosis with normal thyrotropin level); 9.Heart failure (Exception: isolated left ventricular heart failure NYHA ≤ II); 10.Known severe renal insufficiency (glomerular filtration rate < 30 ml/min); 11.One of the following within the previous 2 months: severe hypokalemia or hyperkalemia demanding hospital treatment, narcosis or analgosedation, endoscopic procedure of the gastrointestinal tract, abdominal trauma.  **Factors affecting gastrointestinal microbiota：**  1.Any of the following within the last 2 months: gastrointestinal or respiratory tract infection, food intoxication, major epistaxis requiring treatment by a physician; 2.Antibiotic treatment within the last month.  **Drugs and medications：**  1.Any drug abuse; 2.Any regular use (>2 times a week) of the following medications over the last 2 months: opioids, loperamide, inhaled β-agonists or anticholinergics, glucocorticoids (oral or parenteral), tricyclic antidepressants, antihistaminics with systemic anticholinergic effects, metoclopramide, cholinergics, anticholinergics (except for PD indication), domperidone, protone pump inhibitors. |
| 1. Hill Burns. 2017 | 1.PD subjects were diagnosed by a movement disorder specialist according to the modified UK Brain Bank criteria.  2.Controls were self-reported as being free of neurodegenerative disease.  None of the patients and controls was genetically related to any other patient or control. Fifty-four case-control pairs were spouses; 143 cases and 76 controls were not connected. | Currently taking probiotics (42 cases), Currently taking antibiotics (8 cases), and Completed antibiotics in past 3 months (24 cases) were excluded. |
| 3.Michela Barichella.2019 | 1.Recruited idiopathic PD patients (n = 193) diagnosed according to UK Brain Bank criteria.  2.Patients were compared to a group of control participants (HC; n = 113) matched by age, nutritional status (body mass index), and geographical area (Italian region of residence) and selected among the spouses of the patients included people accompanying them (community healthy controls) or patients attending the same hospital (hospital controls) for other minor health reasons (ie, minor dermatologic, neurologic, ophthalmologic, or orthopedic disorders). | Exclusion criteria for both cases and HC were ongoing artificial nutrition; chronic inflammatory bowel disease; any type of autoimmune disease; acute inflammatory disease (eg, viral/bacterial infections); history of major gastrointestinal surgery; use of antibiotics, probiotics, corticosteroids, or other immunosuppressant medications in the past month; radio-chemotherapy; severe cognitive deficits that precluded the execution of the evaluations; and advanced-stage therapies (deep brain stimulation, continuous apomorphine, or levodopa duodenal infusion). |
| 4.Velma T.E.Aho.2019 | 1.PD subjects were diagnosed by a movement disorder specialist according to the modified UK Brain Bank criteria.  2.age and sex matched subjects | We tested the first five PCs from the diet PCA, all nutrients, the set of 31 food items used for the PCA, and the use of probiotics. None had a significant effect on any of the three alpha diversity indices (adjusted p > 0.2 for all variables). |
| 5.Tengzhu Ren.2020 | 1.All patients eligible for this study were diagnosed for PD according to the UK Brain Bank criteria.  2.13 age-matched healthy spouses of the recruited patients were enrolled as controls | PD：Patients with situation as follows were exluded: (1) secondary Parkinsonism, (2) history of an unstable medical diseases or other diseases interfering with cognitive function evaluation, (3) history of a neurological or psychiatric illness, (4) PD intracranial surgery treatment and deep brain stimulation (DBS) therapy, (5) serious chronic illnesses (e.g., hyperlipidemia, diabetes, hyperhomocysteinemia, heart failure, gastrointestinal, liver cirrhosis, malignancy, or hematological or autoimmune diseases), (6) intake of probiotics or antibiotics within last three months, and (7) have a fat-rich diet.  CON：The exclusion criteria of heathy controls were as follows: serious chronic illnesses; history of an unstable medical diseases or other diseases interfering with cognitive function evaluation; history of a neurological or psychiatric illness; intake of probiotics or antibiotics within last three months and failure to cooperate and complete the trial |
| 6.Chunxiao Li.2019 | 1.diagnosed with PD according to the diagnostic criteria proposed by the International Parkinson Disease and Movement Disorder Society in 2015 in the First Hospital of Jilin University. To be included in the study, PD patients were required to meet the following criteria: no antibiotic use for at least 3 months prior to the study; no digestive system diseases, such as inflammatory bowel disease, no diseases affecting the liver, gall bladder, or pancreas, and no history of surgery on the digestive tract; non-smoking; no alcohol consumption for at least 2 years prior to the study; no autoimmune disease, such as diabetes; no family history of PD; and age at PD onset >50 years. The patients were on PD medications including carbidopa/levodopa and dopamine agonists when the samples were collected.  2.We also recruited the spouses of the PD patients as healthy controls (n = 39) to minimize the influence of lifestyle factors on the gut microbiota. To ensure a sufficient sample size, we further recruited nine age-matched, healthy controls from the local community. Thus, in total, we recruited 48 healthy controls who met the following criteria: no neurodegenerative diseases; no digestive system diseases, no diseases affecting the liver, gall bladder, or pancreas, and no history of surgery on the digestive tract; no hypertension, diabetes, or immune diseases; no antibiotic use, no proton pump inhibitors use, no cardiological drugs use for at least 3 months prior to the study; non-smoking; and no alcohol consumption for more at least 2 years prior to the study. All subjects were of Han Chinese ethnicity and resided in the northeast region of China. In addition, patients and controls were well-matched in terms of age and BMI. | - |
| 7.Wei Li.2017 | 1.PD was diagnosed according to the UK Brain Bank Criteria by experienced neurologists.  2.HC were citizens who matched the PD group by age and gender. | 1.Exclusion criteria for PD subjects were: (i) atypical or secondary Parkinsonism, (ii) regular use of probiotics or antibiotics within three months prior to sample collection, (iii) active or persistent primary gastrointestinal diseases, or (iv) unstable medical, neurological, or psychiatric illness.  2.Exclusion criteria for HC subjects were: (i) active or persistent primary gastrointestinal disease or neurodegenerative diseases, (ii) unstable medical, neurological, or psychiatric illness, or (iii) regular use of probiotics or antibiotics within 3 months before sample collection. |
| 8.Fang Li.2019 | Healthy control participants were the healthy elders at age above 65 years with no symptoms of Parkinson’s disease and had above 3 points in the nonmotor symptoms questionnaire. | Exclusion criteria for subjects with and without PD were as follows; (1) active or persistent primary disease of gastrointestinal tract (e.g. inflammatory bowel disease); (2) alcohol abuse; (3) B-hypovitaminosis; (4) history of hepatobiliary or pancreatic disease; (5) severe gynecological prolapse (grade III); (6) any of the following conditions within the last 2 months: gastrointestinal or respiratory tract infection, food intoxication, major epistaxis requiring treatment by a physician; antibiotic treatment within the last month; any drug abuse; (7) any regular use (> 2 times a week) of the following medications over the last 2 months: opioids, loperamide, inhaled β-agonists or anticholinergics, glucocorticoids (oral or parenteral), tricyclic antidepressants, antihistaminic with systemic anticholinergic effects, metoclopramide, cholinergic, anticholinergics (except for PD indication), domperidone, proton pump inhibitors. |
| 9.Ai Huey Tan.2021 | The study was approved by the University of Malaya Medical Centre (UMMC) Ethics Committee. All subjects provided written informed consent. Patients with PD (n=104) were consecutively recruited from the UMMC Neurology clinic, together with spouse (n=91) or sibling (n=5) controls free of neurological disorders and living in the same community (to minimize potential confounding by factors such as diet, lifestyle and housing condition). PD diagnosis was assigned by Parkinson's neurologists (SYL and AHT) according to standard clinical diagnostic criteria. | Exclusion criteria for all subjects included: antibiotic use within the preceding three months; probiotic use within the preceding month; long-term care residence; tube feeding; and inability to complete study assessments; additionally, for PD patients: antiparkinsonian medication initiation within the preceding three months or adjustment within the preceding month. |
| 10.Fan Zhang.2020 | According to the Movement Disorder Society Clinical Diagnostic Criteria for Parkinson’s disease (MDS-PD Criteria, 2015) of Xiangyang NO.1 People’s Hospital, the patients (PD, n = 63) were recruited by two motor disorder specialists and diagnosed with primary Parkinson's disease.  Healthy spouses (HS, n = 63) were lived in the same household with PD patients. The healthy controls (HP, n = 74) with similar age (± 5 years) and sex ratio were recruited from Physical Examination Center of same hospital. | Patient exclusion criteria: (1) Parkinsonism-plus syndrome or secondary Parkinsonism syndrome; (2) inflammatory bowel syndrome; (3) psychiatric illness; (3) diabetes, gastrointestinal disease, surgical history or infectious diseases; (4) antibiotics/probiotics used for nearly three months.  Exclusion criteria for spouses and control subjects was as follows: (1) obvious digestive diseases (history of gastrointestinal surgery or severe infection); (2) neurodegenerative disease; (3) use of antibiotics/probiotics for nearly three months; and (4) history of going out of Xiangyang city (> 5 days) in the last six months. |
| 11.Yue Peng.2021 | Patients with PD were diagnosed by a specialist and screened over the age of 60 according to the Parkinson's Association's criteria for PD diagnosis.  Recruit at the same time in our hospital check-up of PD old people as control group. | (1)Use antibiotics, steroids and microecologics within 3 months; (2) Non-traumatic infection, allergy, abnormal immune disease, surgery within 3 months; (3) Abnormal bowel habits; (4) Liver and kidney function impairment, chronic gastrointestinal dysfunction, diarrhea, history of biliary tract infection, enteritis and other gastrointestinal diseases; (5) Psychological and behavioral abnormalities. |
| 12.Franziska Hopfner.2017 | All PD cases had been diagnosed by a movement disorder specialist in our clinic according to the UK Parkinson’s Disease Society Brain Bank Clinical Diagnostic Criteria.  A neurodegenerative disease was ruled out in controls. | Antibiotic use within the last three months, as well as gastrointestinal comorbidities were excluded for all participants. |
| 13.J.R.Bedarf.2017 | To reduce any potential gender effects, we included only male participants in the study. Thirty-one male PD patients (diagnosed according to the UK Brain Bank criteria) were compared to 28 male age-matched non-parkinsonian controls. | Further exclusion criteria were: (1) chronic and inflammatory gastrointestinal diseases including chronic constipation; (2) the use of laxatives or immunosuppressive agents in the past three months; (3) atypical or secondary parkinsonism; while (4) the use of antibiotics in the past three months in principle was an exclusion criterion; however, we included three PD patients and three controls despite the intake of antibiotics for one to three days in a period of 28–34 days prior to feces sampling as the omission of those cases from the analyses did not change any result. |
| 14.Aiqun Lin.2018 | All patients with PD (n = 75) were diagnosed by a movement disorder specialist according to the 2015 Clinical Diagnostic Criteria for Parkinson's disease, from the International Parkinson and Movement Disorder Society, and were receiving the medications to treat PD at the time of this study.  The inclusion criteria for control subjects (n = 45) were as follows: (1) a spouse of a participant in the PD group; (2) no use of probiotics or antibiotics within the last three months; (3) no neurodegenerative disease; and (4) no history of chronic or acute gastrointestinal disorders. | Exclusion criteria for subjects with PD were as follows: (1) secondary Parkinsonism; (2) intake of probiotics or antibiotics within the last three months; (3) chronic and inflammatory gastrointestinal diseases; and (4) an unstable medical, neurological, or psychiatric illness. |
